# MRDagent: iterative and adaptive parameter optimization for stable ctDNA-based MRD detection in heterogeneous samples

**DOI:** 10.1093/bioinformatics/btaf485

**Published:** 2025-09-01

**Authors:** Tianci Wang, Xin Lai, Shenjie Wang, Yuqian Liu, Xiaoyan Zhu, Jiayin Wang

**Affiliations:** School of Computer Science and Technology, Xi’an Jiaotong University, Xi’an, Shaanxi, 710049, China; Shaanxi Engineering Research Center of Medical and Health Big Data, Xi’an Jiaotong University, Xi'an, Shaanxi, 710049, China; School of Computer Science and Technology, Xi’an Jiaotong University, Xi’an, Shaanxi, 710049, China; Shaanxi Engineering Research Center of Medical and Health Big Data, Xi’an Jiaotong University, Xi'an, Shaanxi, 710049, China; Department of Respiratory Medicine, The Second Affiliated Hospital of Xi'an Jiaotong University, No. 157, Xiwu Road, Xincheng District, Xi'an, Shaanxi, 710003, China; School of Computer Science and Technology, Xi’an Jiaotong University, Xi’an, Shaanxi, 710049, China; Shaanxi Engineering Research Center of Medical and Health Big Data, Xi’an Jiaotong University, Xi'an, Shaanxi, 710049, China; School of Computer Science and Technology, Xi’an Jiaotong University, Xi’an, Shaanxi, 710049, China; Shaanxi Engineering Research Center of Medical and Health Big Data, Xi’an Jiaotong University, Xi'an, Shaanxi, 710049, China; School of Computer Science and Technology, Xi’an Jiaotong University, Xi’an, Shaanxi, 710049, China; Shaanxi Engineering Research Center of Medical and Health Big Data, Xi’an Jiaotong University, Xi'an, Shaanxi, 710049, China

## Abstract

**Motivation:**

Minimal residual disease (MRD) as critical biomarker for cancer prognosis and management plays a crucial role in improving patient outcomes. However, detecting MRD via next-generation sequencing-based circulating tumor DNA variant calling remains unstable due to the extremely low variant allele frequency and significant inter- and intra-sample heterogeneity. Although parameter optimization can theoretically enhance the detection performance of variants, achieving stable MRD detection remains challenging due to three key factors: (i) the necessity for individualized parameter tuning across numerous heterogeneous genomic intervals within each sample, (ii) the tightly interdependent parameter requirements across different stages of variant detection workflows, and (iii) the limitations of current automated parameter optimization methods.

**Results:**

In this study, we propose MRDagent, a novel variant detection tool designed specifically for MRD detection. MRDagent incorporates an iterative and self-adaptive optimization framework capable of handling unknown objectives, varying constraints, and highly coupled parameters across stages. A key innovation of MRDagent is the integration of a convolutional neural network-based meta-model, trained on historical data to enable rapid parameter prediction. This significantly enhances computational efficiency and generalization performance. Extensive evaluations on simulated and real-world datasets demonstrate MRDagent’s superior and stable performance, providing an efficient, reliable solution for MRD detection in clinical and high-throughput research applications.

**Availability and implementation:**

MRDagent is freely available at https://github.com/aAT0047/MRDagent.git. The corresponding dataset and software archive are available at Zenodo: https://doi.org/10.5281/zenodo.15458496.

## 1 Introduction

Detection of minimal residual disease (MRD) can effectively identify patients at increased risk of recurrence, enabling timely and targeted clinical interventions (Langerhorst *et al.* 2021, Hou *et al.* 2024). Currently, single nucleotide variants (SNVs) and insertion/deletion mutations (indels) in circulating tumor DNA (ctDNA; tumor-derived DNA fragments circulating in blood), analyzed by next-generation sequencing (NGS), represent the most advanced and promising biomarkers for MRD (Pulsipher *et al.* 2022). Ensuring stable performance of MRD detection assays involves two critical aspects: firstly, achieving uniform variant detection performance across all targeted genomic regions within a single sample; secondly, ensuring consistent sensitivity and specificity across diverse patient samples within the cohort (Stutterheim *et al.* 2012, Ryoo *et al.* 2023, Pott *et al.* 2024). Only with such stability can MRD testing accurately reflect disease dynamics and reliably guide clinical decision-making.

However, achieving these two critical aspects to ensure stable MRD detection remains difficult, as the limit of detection is influenced by factors including uneven read distribution across panel regions, artifacts introduced during library preparation, clonal hematopoiesis (Martín-Arana *et al.* 2025, Pantel and Alix-Panabières 2025), and tumor heterogeneity, especially at extremely low ctDNA concentrations [variant allele frequency (VAF)<0.1%]. These factors further exacerbate both intersample and intrasample heterogeneity. Although previous studies have demonstrated that adjusting user-defined parameters can enhance the performance of detection tools (Ewing *et al.* 2015, Lee *et al.* 2018), achieving stable MRD detection faces three critical challenges:

Firstly, stable MRD detection requires individualized parameter optimization tailored specifically to each genomic interval within every sample. However, manual optimization across large cohorts is labor-intensive and typically exceeds feasible manual workload.

Secondly, stable MRD detection necessitates detection tools to simultaneously achieve exceptionally high sensitivity, to reliably identify true low-frequency somatic mutations (Liu *et al.* 2021, Pereira *et al.* 2021), and stringent specificity, to accurately differentiate genuine tumor-derived variants from sequencing artifacts and nontumor biological signals (Pleyer *et al.* 2024). Achieving this goal requires highly coordinated parameter optimization across closely coupled stages of the detection workflow (e.g. variant-calling and filtering steps) (Olson *et al.* 2015, Krusche *et al.* 2019, Koboldt 2020). Specifically, overly stringent thresholds during the preliminary variant-calling stage can inadvertently exclude genuine complex variants, thereby raise the false-negative rate (FNR) and reduce sensitivity. Conversely, overly permissive thresholds may prevent effective removal of sequencing artifacts during filtering, leading to elevated false-positive rates (FPR) and diminished specificity (Cameron *et al.* 2019, Zook *et al.* 2019). Consequently, manual optimization within this highly coupled scenario is both error-prone and poorly reproducible.

Thirdly, automated parameter optimization for stable MRD detection faces significant technical hurdles. These include differences in optimization objectives between workflow stages (e.g. high sensitivity in the calling stage versus high specificity in the filtering stage), the gradient-free nature of the objectives, which rely solely on evaluated metrics (e.g. sensitivity), and unknown constraint thresholds (e.g. acceptable FNR and FPR values) arising from interdependencies among workflow stages. Existing automated optimization strategies, such as metaheuristic algorithms and automated hyperparameter optimization, either lack adaptability to real-time feedback and complex parameter interactions, often converging prematurely to local optima (McCall 2005, Draa *et al.* 2015, Sibalija 2019), or struggle to simultaneously minimize false negatives (FN) and false positives (FP) within a unified optimization framework (Cowen-Rivers *et al.* 2022, Baratchi *et al.* 2024).

In this study, we propose MRDagent, a novel variant detection tool incorporating a self-adaptive optimization framework to address the three challenges. Firstly, MRDagent tackles the first challenge by segmenting genomic regions of ctDNA samples [Browser Extensible Data (BED) files] and optimizing parameters in a region-specific manner. Secondly, MRDagent employs a feedback-driven, self-adjusting optimization loop capable of maintaining stable performance across regions. Thirdly, MRDagent introduces a hybrid reinforcement-optimization strategy. It first utilizes a Deep Q-Network (DQN) framework (Arulkumaran *et al.* 2017) with an embedded alternating direction method of multipliers (ADMM) module (Ariafar *et al.* 2019, Liu *et al.* 2020). The DQN-based agent dynamically and interactively adjusts constraint thresholds across the variant detection and filtering stages, effectively addressing the issue of unknown and dynamically varying constraint thresholds. Then agent is explicitly designed to differentiate stage objectives—maximizing sensitivity during the calling and specificity during the filtering—ensuring targeted adaptation for each phase. Final, the adjusted constraints and observed results are passed into an embedded ADMM module, which iteratively balances constraint satisfaction and objective function optimization, thereby resolving stagewise parameter coupling and improving global detection performance. A key innovation of MRDagent is the integration of a meta-learning model (Boyd *et al.* 2010, Vettoruzzo *et al.* 2024), trained on historical data comprising sample features and parameter configurations. This model enables rapid, region-aware recommendations for optimal parameter settings across heterogeneous genomic intervals, enhancing adaptability and reducing optimization overhead. Together, these components form a closed-loop, data-driven control system that coordinates local region tuning, global consistency, and cross-stage dependency resolution—achieving efficient, robust, and scalable MRD detection.

## 2 Materials and methods

**Figure 1.**
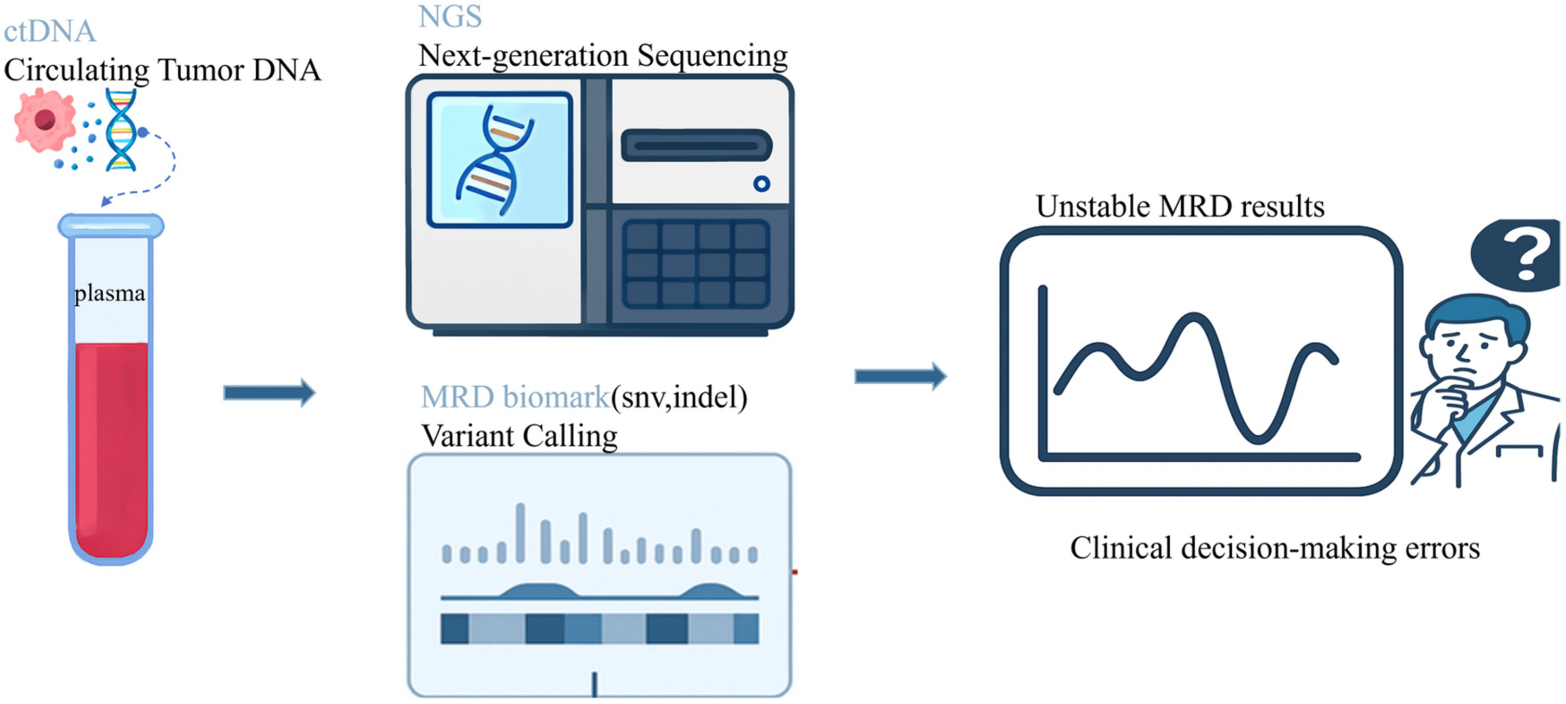
Schematic workflow of ctDNA-based MRD detection using NGS. ctDNA is extracted from plasma samples and subjected to NGS to obtain high-resolution genomic data. Variant calling is then performed to identify MRD-related biomarkers, including SNVs and insertions/deletions (indels). However, due to high tumor heterogeneity and variability in ctDNA levels, MRD results are often unstable, leading to potential errors in clinical decision-making.

As shown in Fig. 1, MRD detection typically uses ctDNA as input, leveraging NGS to obtain high-resolution genomic data, followed by variant calling to identify SNVs and Indels as MRD biomarkers for positive detection. However, the high heterogeneity of tumors and the fluctuating distribution of ctDNA often lead to unstable results, severely affecting clinical decision-making. To address this issue, we propose MRDagent, a robust and efficient algorithm designed to improve detection stability and clinical reliability.

**Figure 2.**
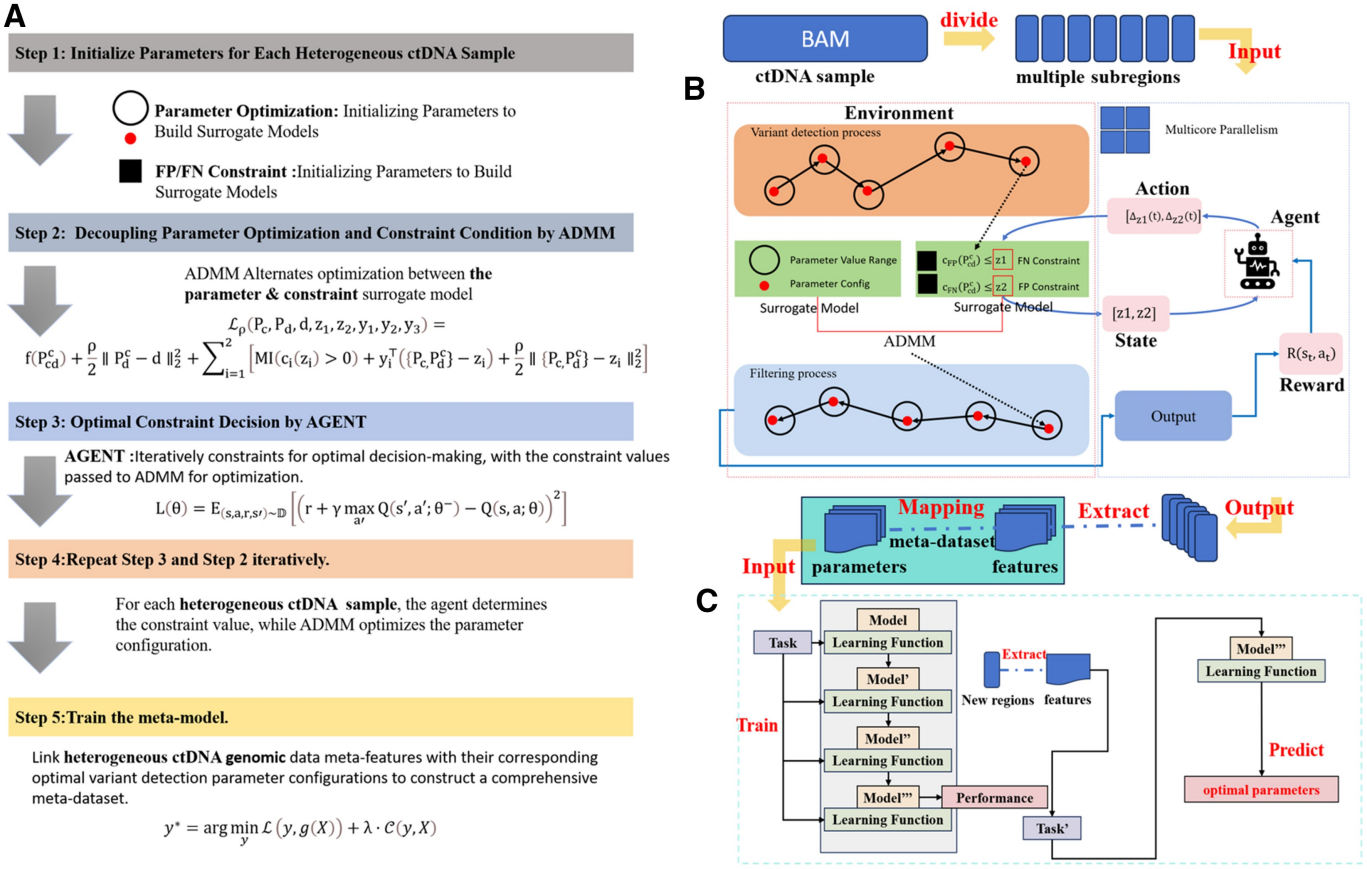
Overall MRDagent workflow diagram. (A) MRDagent consists five key steps: (1) Parameter initialization; (2) decoupling parameters and constraints via alternating direction method of multipliers (ADMM); (3) adaptive constraint adjustment via a deep Q-network (DQN)-based agent; (4) iterative optimization through joint ADMM–DQN processing; (5) meta-model training, which ensures stable and efficient variant detection in heterogeneous ctDNA samples. (B) DQN-based agent in MRDagent dynamically optimizes the threshold values for FPR and FNR constraints. After each ADMM iteration, the agent automatically selects optimal actions based on the current constraints and performance feedback, interactively updating the thresholds for the next iteration. This dynamic adjustment allows for optimal performance under varying sample characteristics and genomic regions. (C) The meta-model, based on CNNs, is trained on historical sample data, including extracted meta-features that capture intersample heterogeneity and corresponding optimal parameter configurations. The meta-model can rapidly and accurately recommend optimal parameters for new ctDNA samples, enhancing the stability, generalization ability, and computational efficiency of the overall detection performance.

Figure 2A illustrates the MRDagent workflow consists of five key steps: 1. Initialize parameters. 2. Decouple parameters and constraints via ADMM. 3. Self-adaptive constraint adjustment via DQN-based agent. 4. Iterative optimization via joint ADMM–DQN processing. 5. Meta-model training.

### 2.1 Initialize parameters

For each panel-sequenced ctDNA sample, we first utilized the BED file [a standard text file format that specifies genomic intervals (regions) targeted by sequencing] to subdivide each sample into multiple subregions using the BEDtools software. Each resulting subregion was then assigned an identical initial parameter configuration (Tables 1 and 2, available as supplementary data at *Bioinformatics* online). Additionally, we set two initial constraint thresholds: the FNR and FPR, both initially set at 0.5, providing a balanced starting point for subsequent adaptive optimization.

### 2.2 Decouple parameters and constraints via ADMM

In MRDagent, the detection-stage objective is high sensitivity, which is computed from discrete counts of true positives (TP), FN, FP, and true negatives (TN) against a gold-standard truth [Variant Call Format (VCF) files]. As such, it cannot be expressed as a continuous and differentiable loss function, nor optimized via gradient-based methods. Moreover, excessively high sensitivity generates a large volume of difficult-to-filter FP, while overly low sensitivity leads a large volume of FN.

Therefore, threshold constraints on the FNR and FPR must be imposed. However, these constraints are typical black-box functions: they are influenced by multiple opaque factors—sequencing quality, alignment algorithms, genomic-interval characteristics, and downstream filtering heuristics—have no explicit analytical form, and can only be evaluated by “set threshold → execute the full pipeline → observe the resulting rate.”

To jointly optimize these gradient-free objectives under black-box constraints, MRDagent employs the ADMM) to decouple the gradient-free objective from the black-box constraints: auxiliary variables split the problem into two subproblems (Methods 1, available as supplementary data at *Bioinformatics* online):

I. Optimality subproblem where sensitivity maximization is solved via Bayesian Optimization (BO) for optimizing the caller parameter configuration.
II. Feasibility subproblem enforcing the black-box constraints.

During iterative updates, ADMM uses Lagrange multipliers and quadratic penalty terms to shuttle information between the subproblems, enabling synchronous convergence of the gradient-free objective and black-box constraints without any explicit constraint models or gradient computations.

The optimization problem is defined as


$$\underset{P_{c},P_{d}}{\min}f(P_{c},P_{d})\;$$

s. t.

$$\;\;c_{\mathrm{FP}}(P_{c},P_{d})\leq 0,c_{\mathrm{FN}}(P_{c},P_{d})\leq 0,$$

$$P_{c}\in C_{ij},\;P_{d}\in D_{ij},\;\;\;\;\;\;\;(1)$$

where f represents the objective function, defined as minimizing the detection error; $c_{\mathrm{FP}}$, $c_{\mathrm{FN}}\;$are black-box constraints for FPR and FNR, respectively, approximated using surrogate models. $P_{c}$ denotes the set of continuous parameters with values from $C_{ij}$, while $P_{d}$ represents the set of discrete parameters with values from $D_{ij}$.

#### 2.2.1 Optimality subproblem

Even within a single stage (detection or filtering), the parameters remain highly coupled, we therefore must jointly optimize the entire parameter set. However, this set contains both discrete and continuous parameters, which cannot be directly handled by a single BO step (Liu *et al.* 2020). To this end, we relax all discrete parameters into a continuous space$\;\tilde{D_{ij}}$. We replace $P_{d}$ with a continuous parameter$\;\tilde{P_{d}}$, introduce a closed-form Euclidean projection constraint, and define an auxiliany variable d. We then construct the augmented Lagrangian function associated with this projection constraint, as follows:


$$\min f(P_{c},\tilde{P_{d}})+\frac{\rho }{2}∥\tilde{P_{d}}-d{∥}_{2}^{2}$$

s. t.

$$c_{\mathrm{FP}}(P_{c},\tilde{P_{d}})\leq 0,\;c_{\mathrm{FN}}(P_{c},\tilde{P_{d}})\leq 0,$$

(2) $$P_{c}\in C_{ij},\tilde{P_{d}}\in \tilde{D_{ij}},$$

where ρ is the penalty parameter, f is gradient-free, we solve this subproblem via BO. After BO get the final $\tilde{P_{d}}$, it is mapped back to the original integer set${\;D}_{ij}$, e.g. via a closed-form Euclidean projection or nearest-neighbor rounding—to ensure that the final parameter values lie in the valid discrete domain.

#### 2.2.2 Feasibility subproblem

We further address the parameter optimization problem with black-box constraints. This subproblem aims to optimize the continuous parameters $\left(P_{c},\tilde{P_{d}}\right)$ to satisfy the black-box constraints of FPR and FNR, while minimizing the objective function $f(P_{c},\tilde{P_{d}}).$ To achieve this, auxiliary variables for each constraint:


(3) z1=cFP(Pc,Pd∼);z2=cFN(Pc,Pd∼).

Based on the ADMM framework, the augmented Lagrangian function $L_{\rho }$for the black-box functions is constructed as follows:


(4) Lρ(Pc,Pd∼,d,z1,z2,y1,y2)=f(Pc,Pd∼)+ρ2∥Pd∼-d∥22+∑i=12[MI(ci(zi)>0)+yi⊤((Pc,Pd∼)-zi)+ρ2∥(Pc,Pd∼)-zi∥22],

where $y_{1}$ and $y_{2}$ denote the Lagrange multipliers (dual variables) associated with the constraints, respectively. M is a sufficiently large penalty constant.

### 2.3 Adaptive constraint adjustment via DQN

In our previous work, we implemented parameter optimization with black-box constraints within an ADMM framework by using static initial thresholds. However, in practical applications, constraints vary dynamically with changes in sample distributions and genomic region characteristics. Consequently, a single, fixed initial threshold struggles to effectively accommodate this dynamic balance.

As show in Fig. 2B, we incorporated the DQN agent into the ADMM optimization framework to dynamically adjust the constraint thresholds for FPR ($c_{\mathrm{FP}}$) and FNR $\left(c_{\mathrm{FN}}).[\mathrm{AQ}9\right]$ After each ADMM iteration, the agent automatically selects actions based on the current state (thresholds) and performance feedback, updating thresholds for the subsequent iteration (Methods 2, available as supplementary data at *Bioinformatics* online):


$$s_{t}=[c_{\mathrm{FP}}(t),c_{\mathrm{FN}}(t)],$$

(5) $$a_{t}=[{\Delta c}_{\mathrm{FP}}(t),{\Delta c}_{\mathrm{FN}}(t)].$$

The thresholds are updated as follows:


$$c_{\mathrm{FP}}(t+1)=c_{\mathrm{FP}}(t)+{\Delta c}_{\mathrm{FP}}(t),$$

(6) $$c_{\mathrm{FN}}(t+1)=c_{\mathrm{FN}}(t)+{\Delta c}_{\mathrm{FN}}(t).$$

Through iterative learning, the DQN agent adaptively optimizes these constraints, achieving optimal performance under varying data conditions. This approach enables interactive adaptation of input–output results between detection and filtering stages and achieves adaptive threshold constraints.

### 2.4 Meta-model

After Sections 2.1–2.3, we obtained optimized parameter configurations for all genomic subregions [Binary Alignment/Map (BAM) ctDNA files] of each ctDNA sample.

#### 2.4.1 Training data

Specifically, as shown in Fig. 2C, we viewed parameter recommendation for each genomic region as distinct yet related “tasks.” For each task, we extracted meta-features X (Table 3, available as supplementary data at *Bioinformatics* online) capturing intersample heterogeneity from corresponding BAM files and combined these with the optimal parameter configurations y, thus forming a meta-dataset.

#### 2.4.2 Model structure

The convolutional neural network (CNN)-based meta-learning model was then trained on this dataset, explicitly learning the common mapping between meta-features and optimal parameters across tasks (Methods 3, available as supplementary data at *Bioinformatics* online):


(7) y*=argminy[ L(y, g(X)) + λ⋅C(y,X)],

where $y^{*}$ represents the optimal predictive structure. y denotes a candidate predictive structure. X is the input data. g(X) is the feature representation. L(y,g(X)) denotes the loss function. C(y,X) is a regularization term. λ is a hyperparameter.

#### 2.4.3 Training process

Finally, we used a 10-fold cross-validation approach to rigorously evaluate the CNN-based meta-model, enabling rapid generalization from previous tasks across samples and genomic regions to new tasks.

This meta-learning model is capable of rapidly inferring optimal parameters for new tasks by leveraging prior task experiences. When handling new heterogeneous ctDNA samples, instead of extensive parameter tuning, the samples are partitioned into multiple subregions. By extracting meta-features that represent local heterogeneity from each subregion, the model efficiently and accurately recommends optimal parameters tailored to each subregion, thus substantially enhancing the stability and generalization of the overall detection performance.

## 3 Results

In order to evaluate our method, we performed the following experiments: (i) we simulated ctDNA samples with VAFs ranging from 0.01% to 0.1%, and assessed MRDagent’s performance (precision and recall). (ii) We utilized real-world data from the International Cancer Genome Consortium, mixed into the human reference genome GRCh38H at VAF levels ranging from 0.01% to 0.1%, to simulate realistic tumor-normal mixture levels. We then evaluated MRDagent’s stability both interheterogeneous and intraheterogeneous (genomic features and VAF distributions). (iii) We explored the capability of MRDagent to dynamically optimization sensitivity and specificity through self-adaptive constraint tuning and dynamic threshold. (iv) Finally, we examined the meta-learning module’s ability to rapidly recommend optimal parameter configurations for new samples, assessing its generalizability and efficiency.

We compare the performance of MRDagent with eight commonly used variant detection tools [Mutect2 (Benjamin *et al.* 2019), Freebayes (Garrison *et al.* 2012), LoFreq (Wilm *et al.* 2012), VarScan2 (Koboldt *et al.* 2012), Bcftools (Danecek *et al.* 2021), SiNVICT (Kockan *et al.* 2017), PACT (Webster *et al.* 2023), and Platypus (Rimmer *et al.* 2014)] (Methods 6, available as supplementary data at *Bioinformatics* online).

To ensure reliable and stable MRD detection, our evaluation strategy clearly distinguishes between two categories of metrics: those evaluating detection performance (precision, recall, F1 score), and those evaluating the stability and consistency of this performance across heterogeneous genomic regions [mean, standard deviation, coefficient of variation (CV), root mean square error (RMSE)] (Methods 4, available as supplementary data at *Bioinformatics* online).

MRDagent consistently outperformed these tools across all experimental scenarios. Furthermore, our results demonstrated that MRDagent exhibited substantially higher stability across intrasamples and genomic regions (intersample), as well as enhanced generalizability capability, such as the meta-learning component of MRDagent rapidly provided optimal parameters to every heterogeneous genomic region, ensuring stable MRD detection results.

### 3.1 Variation detection on simulated data

We first employed 400 simulated ctDNA datasets (Methods 5, available as supplementary data at *Bioinformatics* online) to compare the performance of MRDagent with eight tools (Methods 6, available as supplementary data at *Bioinformatics* online).

**Figure 3.**
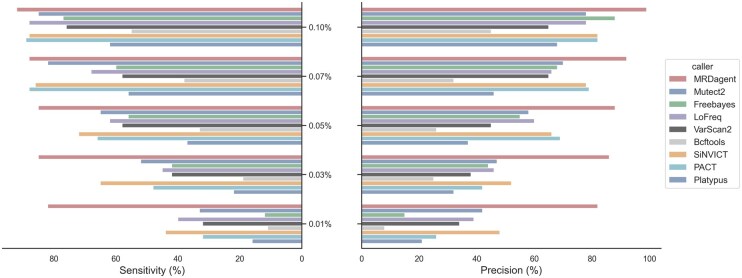
Performance comparison of MRDagent with other variant callers across different VAFs. The sensitivity (left panel) and precision (right panel) are evaluated for MRDagent and eight other tools (Mutect2, Freebayes, LoFreq, VarScan2, Bcftools, SiNVICT, PACT, and Platypus). MRDagent consistently demonstrates superior sensitivity and precision across all tested VAF levels (0.10%, 0.07%, 0.05%, 0.03%, and 0.01%), highlighting its robustness and stability in detecting variants in highly heterogeneous ctDNA samples.

MRDagent demonstrated high sensitivity on this simulated dataset (Fig. 3): it consistently achieved sensitivities exceeding 85% at VAFs of 0.1%, 0.07%, and 0.05%. At lower VAFs (0.03% and 0.01%), MRDagent maintained sensitivities of approximately 83% and 80%, respectively. With respect to specificity, MRDagent again displayed excellent results, consistently exceeding 90% for VAFs≥0.03%. In all cases, MRDagent exhibited higher precision compared to others, and in all tested scenarios, MRDagent’s sensitivity was equal to or greater than these comparison tools.

### 3.2 Variation detection on real-word data

To further demonstrate how MRDagent leverages self-adaptive parameters’ optimization to achieve stable variant detection in heterogeneous ctDNA samples, we performed a comprehensive analysis of 473 subsamples derived from the PACA-CA project (Methods 5, available as supplementary data at *Bioinformatics* online). Firstly, by constructing a sample-mapping relationship (linking the initial 27 samples in Supplementary Table 4 to the resulting 473 subsamples in Tables 2 and 3, available as supplementary data at *Bioinformatics* online), we identified two primary sources of ctDNA heterogeneity: (i) pronounced differences in VAFs across genomic regions, ranging from 0.01% to 0.1% and (ii) substantial variations in mutation types, sizes, and densities across and within samples. Based on this mapping, we evaluated the effects of both intersample and intrasample heterogeneity on the performance of various variant detection tools.

#### 3.2.1 Analysis of feature heterogeneity

**Figure 4.**
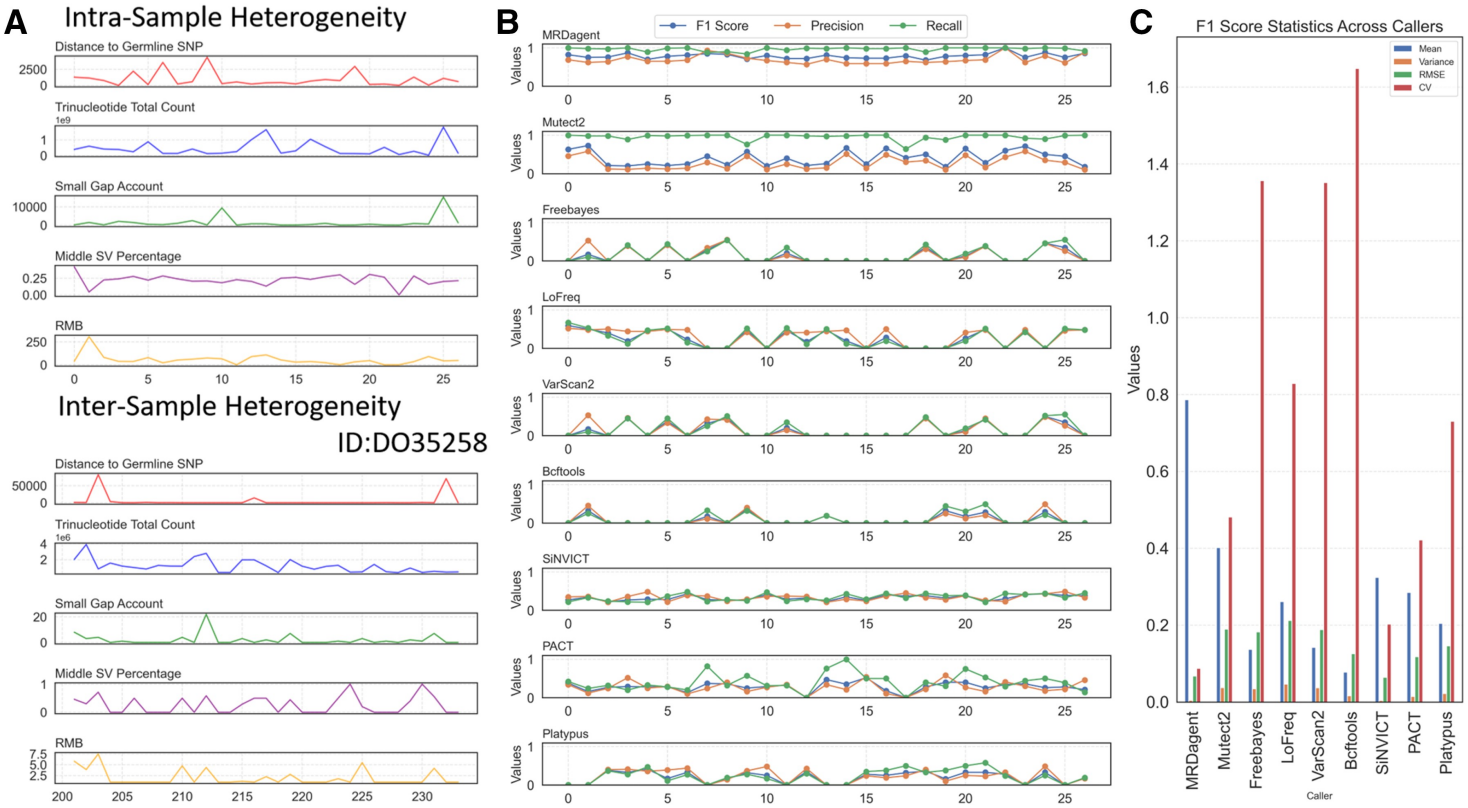
Evaluation of MRDagent performance under intrasample and intersample heterogeneity. (A) Metrics illustrating heterogeneity characteristics, including distance to germline SNPs, trinucleotide count, small gap count, middle SV percentage, and read mismatch bias (RMB). These features capture intrasample and intersample variability, with the “Distance to Germline SNP” helping refine filtering thresholds, and “Small Gap Count” aiding in InDels detection. The “Middle SV Percentage” and “Large SV Percentage” reflect structural variant distribution, highlighting the need to adjust breakpoint thresholds. (B) Comparison of F1 score, precision, and recall for MRDagent and other variant callers (Mutect2, Freebayes, LoFreq, VarScan2, Bcftools, SiNVICT, PACT, and Platypus) across heterogeneous samples. MRDagent shows more consistent performance. (C) Statistical analysis of F1 scores, including mean, variance, RMSE, and CV, demonstrating MRDagent’s superior stability and robustness under heterogeneous conditions.

We first merged the 473 subsamples back into the original 27 samples and extracted key features from each (see Section 2). As illustrated in Fig. 4A, the five features with the greatest variance exhibit pronounced fluctuations both between samples (27 original samples) and within individual samples (e.g. sample ID: DO35258, mapped indices 201–234). These findings suggest significant feature heterogeneity that must be carefully addressed to improve the precision of variant identification and classification (Table S3, available as supplementary data at *Bioinformatics* online).

#### 3.2.2 Stability analysis of intersample samples

As shown in Fig. 4B, MRDagent demonstrated exceptional stability across all 27 samples, successfully detecting MRD status in all cases (27 out of 27). The lowest VAF successfully detected by MRDagent was 0.01%. Although SiNVICT also successfully identified MRD in all 27 patients and showed relative stability across samples, its stringent parameter thresholds and multilevel filtering resulted in low sensitivity, maintaining an F1 score around 0.5. Yet, other tools exhibited instability due to their default or manually set parameters inadequately adapting to the heterogeneous characteristics of ctDNA samples. Specifically, LoFreq achieved high F1 scores (>0.8) in only 4 of 27 samples (∼15%). Freebayes, VarScan2, and Bcftools exhibited similar instability, with their F1 scores frequently falling below 0.2 in over half of the samples (≥14 of 27 samples). Furthermore, PACT and Platypus displayed considerable performance volatility, with most samples (≥20 out of 27) showing low F1 scores (<0.4).

#### 3.2.3 Stability analysis of intrasample samples

As illustrated in Fig. 4C, MRDagent attained a mean F1 score of 0.78 across the 473 subsamples, with a variance of merely 0.04, and notably lower RMSE and CV compared to other methods. These statistical indicators confirm stability of MRDagent, reflected in its consistently high detection performance across diverse ctDNA samples. Conversely, Bcftools, Freebayes, LoFreq, VarScan2, and PACT showed considerably higher CV values, indicative of substantial performance instability. While SiNVICT exhibited relatively lower variation in F1 scores, its mean F1 score around 0.4.

### 3.3 Dynamic constraints for optimizing MRD detection sensitivity and specificity

Variant detection tools generally consist of integrated mathematical models and algorithms, each highly dependent on various threshold values which are parameters. Given the inherent heterogeneity among ctDNA samples, these parameters must be dynamically optimized according to sample-specific characteristics to achieve stable and accurate detection performance. In preliminary variant calling aims at enhancing sensitivity to maximize TPs, while subsequent filtering seeks to reduce FPs for improved accuracy. These two stages present a significant tradeoff: overly relaxed thresholds produce numerous FPs difficult to filter, while overly stringent thresholds miss TP variants. Consequently, introducing specific constraints during parameter optimization is necessary to dynamically balance sensitivity and specificity. Due to pronounced sample heterogeneity, these constraints must adapt dynamically for each sample.

#### 3.3.1 Experimental design

To clearly illustrate the roles of MRDagent’ constraints and dynamic threshold adjustments, we designed three experiments:

Experiment 1: BO minimizes FNR during preliminary calling and FPR during filtering, evaluating final F1 scores.

Experiment 2: BO maximizes F1 scores directly in both preliminary calling and filtering stages.

Experiment 3: MRDagent dynamically optimizes parameters using a DQN framework, interactively balancing sensitivity and specificity between the two stages.

#### 3.3.2 Comparative analysis of performance

**Figure 5.**
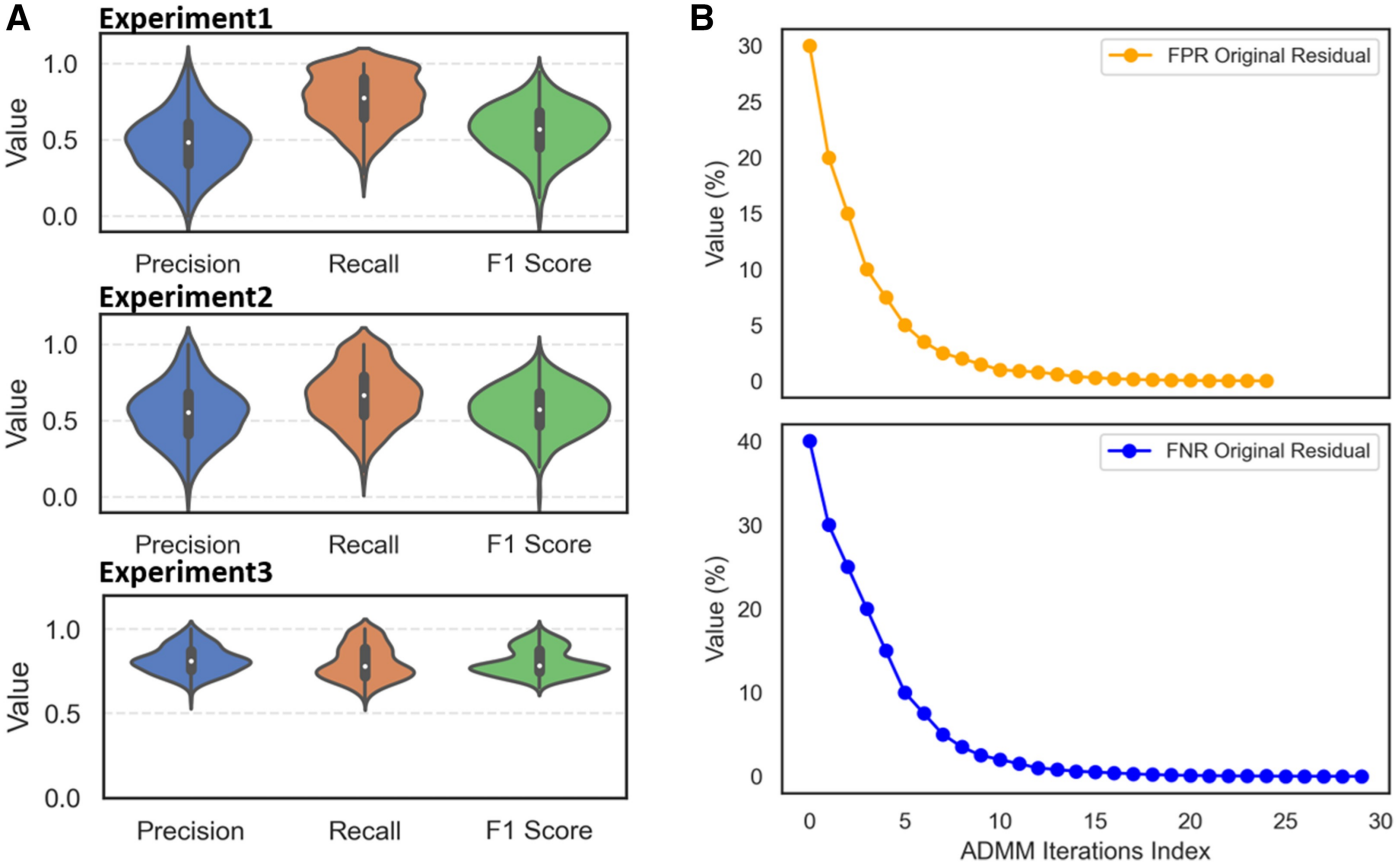
Performance comparison across different experimental setups and ADMM optimization progress. (A) Violin plots showing the distribution of precision, recall, and F1 scores across three experimental setups: Experiment 1, Experiment 2, and Experiment 3. Experiment 1, which minimizes FNR and FPR independently, exhibits a significant gap between precision and recall, resulting in lower F1 scores. Experiment 2, where both stages optimize F1 directly, shows improved balance but with limited gains in F1 performance. Experiment 3, employing MRDagent with dynamic constraint adjustment and the ADMM framework, demonstrates superior precision, recall, and F1 scores, with reduced variability across metrics. (B) Convergence trends of the ADMM optimization process. The top panel illustrates the reduction of the original residual for the FPR, while the bottom panel shows the corresponding residual trend for the FNR. Both residuals rapidly decrease and stabilize at low levels within a small number of ADMM iterations, indicating the satisfaction of constraints and convergence of the optimization process.

As illustrated in Fig. 5A, Experiment 1 separately optimizes FNR and FPR, effectively reducing missed detections but introducing many difficult-to-filter FP, resulting in low overall F1 scores. Experiment 2 directly targets F1 maximization, moderately improving performance by balancing errors but still constrained by coupled parameters, resulting in limited gains. In Experiment 3, however, dynamically adjusts parameter thresholds using DQN combined with ADMM, achieving significantly higher precision, recall, and F1 scores, alongside excellent stability.

Figure 5B shows the original residual trends for FPR and FNR during the ADMM process. Residual values rapidly decrease and stabilize at low levels as iterations proceed, indicating successful constraint satisfaction. In summary, MRDagent’s interactive parameter optimization effectively resolves the tradeoff between preliminary detection and filtering, delivering consistently accurate and stable variant detection outcomes under constrained conditions.

### 3.4 Meta-model is needed for rapid parameter configuration

As previously described, MRDagent’s iterative interactions between the agent and ADMM module result in exponentially increased computational complexity. Moreover, due to the absence of an explicit loss function for gradient calculation, parameter optimization relies solely on evaluation against a gold-standard reference, significantly restricting its applicability to new ctDNA samples.

**Figure 6.**
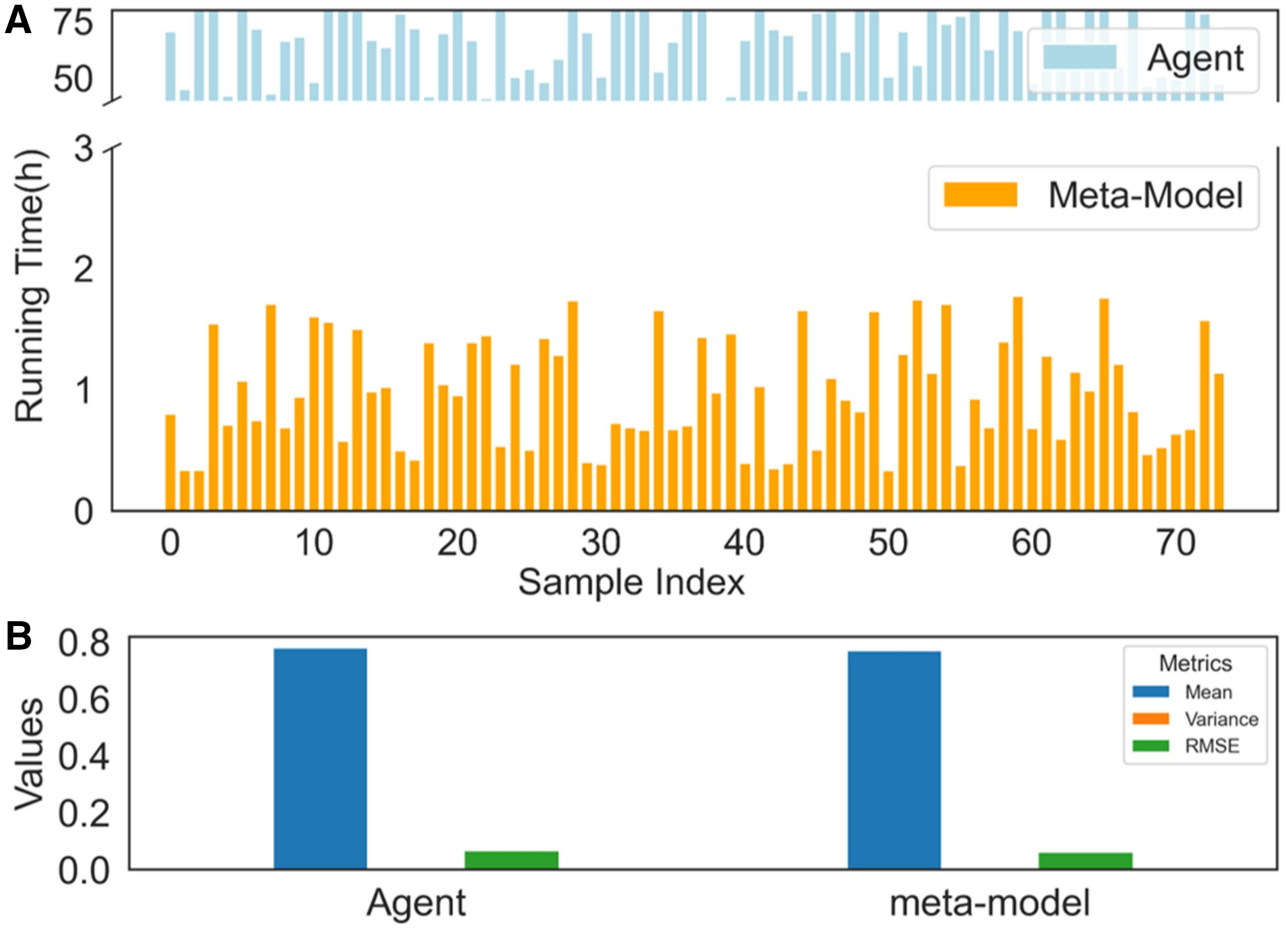
Comparison of the agent and meta-model approaches in terms of runtime and performance metrics. (A) Runtime comparison between the agent and meta-model across 74 test samples. The agent requires significantly longer execution times due to iterative optimization during parameter tuning. In contrast, the meta-model achieves rapid parameter configuration by leveraging pretrained mappings of sample features to parameter settings, resulting in much shorter runtimes. (B) Performance evaluation of the agent and meta-model using three key metrics: mean, variance, and RMSE of the F1 score. Both methods exhibit similar mean F1 scores, highlighting the meta-model’s ability to approximate the agent’s performance effectively. Additionally, the meta-model demonstrates low variance and RMSE, indicating consistent and stable performance across samples. We trained a CNN-based meta-model using historical samples (X: Table 3, available as supplementary data at *Bioinformatics* online; y: Table 5, available as supplementary data at *Bioinformatics* online), We conducted 10-fold cross-validation on 474 samples, using 427 for training and 47 for testing in each fold.

As shown in Fig. 6A, the CNN meta-model achieves substantially faster inference compared to the reinforcement learning-based agent. Figure 6B compares mean F1 score, variance, and RMSE on the test set. The CNN model closely matches agent performance, with an F1 difference <0.01 and comparable stability, indicating strong generalization capability. Consequently, these results demonstrate that the CNN meta-model offers fast and reliable parameter recommendations for heterogeneous ctDNA samples without requiring gold-standard results, making it well suited for large-scale, high-throughput variant detection applications.

## 4 Discussion 

MRDagent is a stable variant detection tool that integrates DQN-based reinforcement learning and ADMM optimization, designed specifically to dynamically adapt to ctDNA sample heterogeneity and achieve stable MRD detection. Unlike conventional tools that rely on fixed parameter thresholds, MRDagent’s primary advantage lies in its ability to automatically adjust all parameters based on sample-specific characteristics. Additionally, MRDagent efficiently generalizes through a CNN-based meta-model, which enables it to adapt to a wide range of sample variations. Experimental results demonstrate that MRDagent consistently achieves higher and more stable F1 scores (mean = 0.78, variance = 0.04) across both 473 subsamples and 27 highly heterogeneous samples within the VAF range of 0.01%–0.1%. These results highlight the method's stability in handling ctDNA samples with varying heterogeneity. Furthermore, MRDagent outperforms other methods in terms of computational efficiency, providing faster and more reliable results compared to existing approaches.

## 5 Conclusion

In summary, MRDagent offers a stable and practical solution for stable and efficient MRD detection in heterogeneous ctDNA samples. Its ability to adjust parameters dynamically, combined with the power of reinforcement learning and ADMM optimization, enables it to handle the challenges posed by sample heterogeneity effectively. The tool’s superior performance, as demonstrated by its high and stable F1 scores and improved computational efficiency, underscores its potential to advance MRD detection in clinical applications.

## Supplementary Material

btaf485_Supplementary_Data

## Data Availability

The data underlying this article were accessed from the International Cancer Genome Consortium (ICGC, https://dcc.icgc.org/). Access to the original data requires compliance with the ICGC Data Access Policies and approval by the relevant Data Access Committee. The derived data generated in this study are available from the corresponding author upon reasonable request.
