# Supplementary material for: MRDagent: iterative and adaptive parameter optimization for stable ctDNA-based MRD detection in heterogeneous samples
Source: Bioinformatics. 2025 Sep 1;41(9):btaf485. doi: 10.1093/bioinformatics/btaf485 (PMC12462387; doi:10.1093/bioinformatics/btaf485)
Supplement: btaf485_Supplementary_Data [file btaf485_supplementary_data.zip › Supplementary_Methods.docx]

**MRDagent: Iterative and Adaptive Parameter Optimization for stable ctDNA-Based MRD Detection in Heterogeneous Samples**

**Supplementary Methods**

1. **Alternating Direction Method of Multipliers (ADMM) module**

Definition of Optimization Problem

This study aims to solve the following constrained optimization problem:

$$\begin{aligned} \begin{matrix} \min_{P_{c},P_{d}} & f\left( P_{c},\tilde{P_{d}} \right)+\frac{\rho}{2}\parallel\tilde{P_{d}}-d\parallel_{2}^{2}, \\ \text{s.t.} & c_{TP}\left( P_{c},\tilde{P_{d}} \right)\leq0, \\ & c_{FN}\left( P_{c},\tilde{P_{d}} \right)\leq0, \\ & P_{c},\tilde{P_{d}}\in C_{ij}+D_{ij}^{c}, \end{matrix}\#\left( 1 \right) \end{aligned}$$

where $f\left( P_{c},\tilde{P_{d}} \right)$ is the objective function aimed at minimizing error or cost. The constraints $c_{TP}\left( P_{c},\tilde{P_{d}} \right)$ and $c_{FN}\left( P_{c},\tilde{P_{d}} \right)$ represent the true positive rate (TPR) and false negative rate (FNR), respectively, treated as black-box functions. $P_{c}$ and $P_{d}$ are continuous parameter sets and relaxed continuous-integer parameter sets, constrained within $C_{ij}$ and $D_{ij}^{c}$. The auxiliary variable $d$ is introduced to handle Euclidean projection constraints, and $\rho$ is a penalty parameter balancing the objective function and penalty terms.

Reformulation with Auxiliary Variables and Indicator Functions

To convert inequality constraints into equality constraints and penalize constraint violations effectively, auxiliary variables and indicator functions are introduced. Specifically, the auxiliary variables $z_{1}$ and $z_{2}$ are defined as:

$$\begin{aligned} z_{1}=c_{TP}\left( P_{c},\tilde{P_{d}} \right), z_{2}=c_{FN}\left( P_{c},\tilde{P_{d}} \right),\#\left( 2 \right) \end{aligned}$$

and the indicator function $I\left( \cdot\right)$ is defined as:

$$\begin{aligned} I\left( \text{condition} \right)=\left\{ \begin{matrix} 1 & \text{if condition is true,} \\ 0 & \text{otherwise.} \end{matrix} \right.\#\left( 3 \right) \end{aligned}$$

A large constant $M$ is introduced to heavily penalize constraint violations, thereby approximating the original constrained optimization problem. Based on the Alternating Direction Method of Multipliers (ADMM) framework, the augmented Lagrangian function $\mathcal{L}_{\rho}$ is constructed as:

$$\mathcal{L}_{\rho}\left( P_{c},P_{d},d,z_{1},z_{2},y_{1},y_{2},y_{3} \right)=f\left( P_{c},\tilde{P_{d}} \right)+\frac{\rho}{2}$$

$$\begin{aligned} \parallel P_{d}^{c}-d\parallel_{2}^{2}+\sum_{i=1}^{2} \left[ MI\left( c_{i}\left( z_{i} \right)>0 \right)+y_{i}^{\top}\left( \left( P_{c},\tilde{P_{d}} \right)-z_{i} \right)+\frac{\rho}{2}\parallel\left( P_{c},\tilde{P_{d}} \right)-z_{i}\parallel_{2}^{2} \right]\#\left( 4 \right) \end{aligned}$$

where $y_{1}$ and $y_{2}$ are dual variables (Lagrange multipliers) corresponding to the constraints $c_{TP}\left( P_{c},\tilde{P_{d}} \right)\leq0$ and $c_{FN}\left( P_{c},\tilde{P_{d}} \right)\leq0$, respectively.

ADMM Iteration Steps

The ADMM optimization procedure consists of the following main steps:

Step 1: Update Primal Variables $P_{c}$ and $P_{d}$

With auxiliary variables $d$, $z_{1}$, $z_{2}$, and dual variables $y_{1}$, $y_{2}$, $y_{3}$ fixed, the primal variables $P_{c}$ and $P_{d}$ are updated by solving:

$$\begin{aligned} \left( P_{c}^{k+1},P_{d}^{k+1} \right)=arg\min_{P_{c},P_{d}}\mathcal{L}_{\rho}\left( P_{c},P_{d},d^{k},z_{1}^{k},z_{2}^{k},y_{1}^{k},y_{2}^{k},y_{3}^{k} \right).\#\left( 5 \right) \end{aligned}$$

Since $c_{TP}$ and $c_{FN}$ are black-box functions with no explicit gradients, optimization is performed using the following methods:

- Surrogate Models: Approximate $c_{TP}$ and $c_{FN}$ using Gaussian Process Regression or other machine learning techniques, and optimize based on the surrogate models’ predictions.
- Heuristic Methods: Employ heuristic algorithms such as Genetic Algorithms or Particle Swarm Optimization to optimize $P_{c}$ and $P_{d}$, while evaluating the feasibility of constraints during the process.

Step 2: Update Auxiliary Variables $d$, $z_{1}$, and $z_{2}$

With $P_{c}$, $P_{d}$, and dual variables fixed, the auxiliary variables are updated as:

$$d^{k+1}=P_{d}^{k+1}-\frac{y_{1}^{k}}{M},$$

$$z_{1}^{k+1}=min\left( c_{TP}\left( \left( P_{c},\tilde{P_{d}} \right) \right)^{k+1}-\frac{y_{2}^{k}}{M}, 0 \right),$$

$$\begin{aligned} z_{2}^{k+1}=min\left( c_{FN}\left( \left( P_{c},\tilde{P_{d}} \right) \right)^{k+1}-\frac{y_{3}^{k}}{M}, 0 \right),\#\left( 6 \right) \end{aligned}$$

ensuring $z_{1}\leq0$ and $z_{2}\leq0$ through projection operations.

Step 3: Update Dual Variables $y_{1}$, $y_{2}$, and $y_{3}$

The dual variables are updated based on the differences between the primal and auxiliary variables:

$$y_{1}^{k+1}=y_{1}^{k}+M\left( d^{k+1}-P_{d}^{k+1} \right),$$

$$y_{2}^{k+1}=y_{2}^{k}+M\left( z_{1}^{k+1}-c_{TP}\left( \left( P_{c},\tilde{P_{d}} \right) \right)^{k+1} \right),$$

$$\begin{aligned} y_{3}^{k+1}=y_{3}^{k}+M\left( z_{2}^{k+1}-c_{FN}\left( \left( P_{c},\tilde{P_{d}} \right) \right)^{k+1} \right).\#\left( 7 \right) \end{aligned}$$

Step 4: Convergence Criteria

At each iteration, the primal and dual residuals are computed to assess convergence:

$$\text{Primal Residual}=\parallel d^{k+1}-P_{d}^{k+1}\parallel_{2},$$

$$\text{Dual Residual}_{z_{1}}=\parallel z_{1}^{k+1}\parallel_{2},$$

$$\begin{aligned} \text{Dual Residual}_{z_{2}}=\parallel z_{2}^{k+1}\parallel_{2}.\#\left( 8 \right) \end{aligned}$$

If all residuals fall below a pre-defined threshold $\epsilon$, the algorithm is considered converged; otherwise, the iterations continue.

Theoretical Equivalence

As discussed in Boyd et al. (2011), when $M$ is sufficiently large, the indicator function and penalty terms impose heavy penalties on constraint violations, ensuring that only feasible solutions are considered. Consequently, the unconstrained optimization problem is mathematically equivalent to the original constrained optimization problem. The dual variables $y_{i}$ facilitate the alignment of primal and auxiliary variables, steering the optimization process toward constraint satisfaction.

Reference

[1] Boyd, S., Parikh, N., Chu, E., Peleato, B., & Eckstein, J. (2011). Distributed Optimization and Statistical Learning via the Alternating Direction Method of Multipliers. Foundations and Trends® in Machine Learning, 3(1), 1–122.

| **Algorithm 1** ADMM Optimization |
| --- |
| $\mathbf{Require}:$  $\bullet Objective function f\left( P_{c},\tilde{P}_{d} \right)$  $\bullet$ Constraint functions $c_{T}P\left( z_{1} \right)$ and $c_{F}N\left( z_{2} \right)$ (black-box)  $\bullet$ Penalty parameter $\rho$  $\bullet$ Large constant $M$  $\bullet$ Indicator function I ($\cdot)$  $\bullet$ Initial values $P_{c}^{0},\tilde{P}_{d}^{0},z_{1}^{0},z_{2}^{0},d^{0}$  $\bullet$ Initial dual variables $y_{1}^{0},y_{2}^{0},y_{3}^{0}$  $\bullet$ Maximum iterations $K$ $\bullet$ Convergence threshold $\epsilon=0.01$  $\mathbf{Ensure}:$  $\bullet$Optimal solution$P_{c}^{*},\tilde{P}_{d}^{*}$  1: Initialize variables：  2: $P_{c}\leftarrow P_{c}^{0}$, $\tilde{P}_{d}\leftarrow\tilde{P}_{d}^{0}$  3: $z_{1}\leftarrow z_{1}^{0},z_{2}\leftarrow z_{2}^{0}$, $d\leftarrow d^{0}$  4: $y_{1}\leftarrow y_{1}^{0},y_{2}\leftarrow y_{2}^{0}$, $y_{3}\leftarrow y_{3}^{0}$  5: Initialize Surrogate Models for $c_{TP}$ and $c_{FN}$  6: Initialize Surrogate Models for $P_{c}^{*}$, $\tilde{P}_{d}^{*}$  7: Initialize datasets $\mathcal{D}_{1}\leftarrow\emptyset,\mathcal{D}_{2}\leftarrow\emptyset$  8: for $k=1$ to $K$ do  9: Update primary variables $P_{c},\tilde{P}_{d}$, and $d:$  $\left( P_{c}^{k+1},\tilde{P}_{d}^{k+1},d^{k+1} \right)=arg\min_{P_{c},\tilde{P}_{d},d}(f\left( P_{c},\tilde{P}_{d} \right)+\frac{\rho}{2}\parallel P_{c}-d+\frac{y_{3}^{k}}{\rho}\parallel_{2}^{2})$  10: Update auxiliary variable $z_{1}:$  $z_{1}^{k+1}=arg\min_{z_{1}} \left( c_{TP}(z_{1})+M\mathbb{I(}z_{1})+\frac{\rho}{2}\parallel P_{c}^{k+1}-z_{1}+\frac{y_{1}^{k}}{\rho}\parallel_{2}^{2} \right)$  11: Evaluate $c_{TP}\left( z_{1}^{k+1} \right)$, add to $\mathcal{D}_{1}$, update BO model  12: Update auxiliary variable$z_{2}$:  $z_{2}^{k+1}=arg\min_{z_{2}} \left( c_{FN}(z_{2})+M\mathbb{I(}z_{2})+\frac{\rho}{2}\parallel P_{c}^{k+1}-z_{2}+\frac{y_{2}^{k}}{\rho}\parallel_{2}^{2} \right)$  13: Evaluate$c_{FN}\left( z_{2}^{k+1} \right)$, add to $\mathcal{D}_{2}$, update BO model  14: Update dual variables:  $\begin{aligned} y_{1}^{k+1}=y_{1}^{k}+\rho(P_{c}^{k+1}-z_{1}^{k+1}), \\ y_{2}^{k+1}=y_{2}^{k}+\rho(P_{c}^{k+1}-z_{2}^{k+1}), \\ y_{3}^{k+1}=y_{3}^{k}+\rho\left( P_{c}^{k+1}-d^{k+1} \right). \end{aligned}$  15: Check convergence:  $\mathrm{prima}l_{\_}esidual=\parallel P_{c}^{k+1}-z_{1}^{k+1}\parallel_{2}+\parallel P_{c}^{k+1}-z_{2}^{k+1}\parallel_{2}+\parallel P_{c}^{k+1}-d^{k+1}\parallel_{2},$  $dual\_esidual=\rho\parallel z_{1}^{k+1}-z_{1}^{k}\parallel_{2}+\rho\parallel z_{2}^{k+1}-z_{2}^{k}\parallel_{2}+\rho\parallel d^{k+1}-d^{k}\parallel_{2}.$  16: if $\mathrm{prima}l_{\_}\mathrm{esidual}$ $<\epsilon$ and dual residual$<\epsilon$ then  17: break  18: end if  19: end for |
| 20: Return$P_{c}^{*}, \tilde{P}_{d}^{*}=P_{c}^{k+1}, \tilde{P}_{d}^{k+1}$ |

1. **Adaptive Constraint Adjustment via DQN**

In earlier work, we incorporated black-box-constrained parameter optimisation into an ADMM framework by employing a fixed initial value. However, in practical applications, the constraints governing detection and filtering often exhibit dynamic equilibrium properties, meaning that the optimal false positive rate and false negative rate thresholds adapt to data-specific characteristics to ensure stable performance. Yet, these dynamic conditions are unknown and cannot be effectively addressed by static initial values. To overcome this limitation, we propose a Deep Reinforcement Learning (DRL)-based agent designed to dynamically determine the optimal black-box constraint values during the parameter-optimisation process.

Agent

By interacting with the environment, the agent learns to adapt the false positive $c_{F}P\left( P_{cd}^{c} \right)\leq0$ and false negative $c_{F}N\left( P_{cd}^{c} \right)\leq0$ constraints at different stages of optimisation, thereby achieving an optimal balance for the overall objective.

State

We define the state $S$ as the current values of the constraint conditions on the false positive rate $\left( \begin{matrix} c_{FP} \end{matrix} \right)$ and false negative rate $\left( \begin{matrix} c_{FN} \end{matrix} \right).$The state vector at iteration $t$ is given by

$$\begin{aligned} s_{t}=\left[ \begin{matrix} c_{FP}\left( t \right), c_{FN}\left( t \right) \end{matrix} \right]\#\left( 9 \right) \end{aligned}$$

where $c_{F}P\left( t \right)$ and $c_{FN}\left( t \right)$ denote the constraint values for the false positive rate and false negative rate at the $t$-th optimisation iteration, respectively. The initial state is set to [0.3,0.3], indicating that the false positive rate should not exceed 0.3, while the false negative rate should remain below 0.3.

Action

The action $A$ is defined as the amount by which the constraints $c_{F}P$ and $c_{FN}$ are adjusted in a single iteration. Specifically, we denote:

$$\begin{aligned} a_{t}=\left[ \begin{matrix} \Delta_{FP}\left( t \right), \Delta_{FN}\left( t \right) \end{matrix} \right], \Delta_{FP}\left( t \right), \Delta_{FN}\left( t \right)\in\left[ -0.05, 0.05 \right]\#\left( 10 \right) \end{aligned}$$

where $\Delta_{F}P\left( t \right)$ and $\Delta_{F}N\left( t \right)$ represent the adjustments made to the false positive rate and false negative rate constraints at iteration $t$, respectively

Reward Function

We define $R_{F1}=F1$, which is obtained by evaluating the filtered VCF file and captures the effectiveness of the detection process. To penalise large adjustments to the constraints, we employ

$$\begin{aligned} R_{\Delta}=-\left( \Delta_{\mathrm{TP}}^{2}+\Delta_{\mathrm{FN}}^{2} \right)\#\left( 11 \right) \end{aligned}$$

thereby encouraging smoother actions. Meanwhile

$$\begin{aligned} R_{\mathrm{balance}}=\frac{2\times min\left( FPC, FNC \right)}{FPC+FNC}\#\left( 12 \right) \end{aligned}$$

quantifies the balance between false positives and false negatives, encouraging the model to maintain an optimal trade-off. We set the weight coefficients to $w_{1}=1.0,w_{2}=0.2$ and $w_{3}=$ -0.3. Hence, the overall reward function is:

$$\begin{aligned} R\left( s_{t},a_{t} \right)=w_{1} R_{\text{F}1} + w_{2} R_{\Delta} + w_{3} R_{\text{balance}}\#\left( 13 \right) \end{aligned}$$

Loss Function

We adopt the standard Deep Q-Network (DQN) loss function

$$\begin{aligned} L\left( \theta\right) = \mathbb{E}_{\left( s,a,r,s^{'} \right)\mathcal{\sim D}}\left[ (r + \gamma\max_{a^{'}}Q\left( s^{'} ,a^{'};\theta^{-} \right) - Q\left( s,a;\theta\right))^{2} \right]\#\left( 14 \right) \end{aligned}$$

where $\mathcal{D}$ is the experience replay buffer, $\gamma$ is the discount factor controlling the significance of future rewards, and $\theta^{-}$ denotes the parameters of the target network, which are periodically updated from the main network $\theta$ to stabilise training

iterative

The agent dynamically adjusts $c_{FP}$ and $c_{FN}$ and then feeds the updated constraint values into the ADMM framework for parameter optimisation. At each iteration $t$, the agent observes the current constraint values $s_{t}=[$ $c_{FP}\left( t \right)$, $c_{FN}\left( t \right)$ ], chooses an action $a_{t}=\left[ \Delta_{\mathrm{FP}}\left( t \right),\Delta_{\mathrm{FN}}\left( t \right) \right]$, and updates the constraints accordingly:

$$\begin{aligned} c_{FP}\left( t+1 \right) = c_{FP}\left( t \right) + \Delta_{\mathrm{FP}}\left( t \right), c_{FN}\left( t+1 \right) = c_{FN}\left( t \right) + \Delta_{\mathrm{FN}}\left( t \right)\#\left( 15 \right) \end{aligned}$$

These revised constraints $\left[ c_{FP}\left( t+1 \right),c_{FN}\left( t+1 \right) \right]$ are then passed to the ADMM framework, which re-optimises the model. Based on the outcome of this optimisation, a reward $r_{t}$ is computed and provided to the agent, guiding the policy update. Through iterative refinement, this process converges on optimal variant detection and filtering performance.

| Algorithm 1 MRDagent |
| --- |
| Require:  $\bullet$Objective function $f\left( P_{c},P_{d} \right)$  $\bullet$ Constraint functions $c_{T}P\left( z_{1} \right)$ and $c_{F}N\left( z_{2} \right)$ (black-box)  $\bullet$ Penalty parameter $\rho$  $\bullet$ Initial values $P_{c}^{0},P_{d}^{0},z_{1}^{0},z_{2}^{0},d^{0}$  $\bullet$ Initial dual variables $y_{1}^{0},y_{2}^{0},y_{3}^{0}$  $\bullet$ Maximum iterations $K$  $\bullet$ Convergence threshold $\epsilon$  $\bullet$ DQN parameters: learning rate, discount factor, exploration rate, etc. $\bullet$ Meta-model parameters: model architecture, training parameters, etc.  $Ensure:$  $\bullet$ Optimal solution $P_{c}^{*},P_{d}^{*}$  $\bullet$ Trained meta-model for parameter recommendation  1: Initialize ADMM Optimization:  2: $P_{c}\leftarrow P_{c}^{0}$, $P_{d}\leftarrow\dot{P_{d}^{0}}$,  3: $z_{1}\leftarrow z_{1}^{0}, z_{2}\leftarrow z_{2}^{0}, d\leftarrow d^{0},$  4: $y_{1}\leftarrow y_{1}^{0}, y_{2}\leftarrow y_{2}^{0}, y_{3}\leftarrow y_{3}^{0}$  5: Initialize Surrogate Model  6: Initialize datasets$\mathcal{D}_{1}\leftarrow\emptyset,\mathcal{D}_{2}\leftarrow\emptyset$  7: Initialize Deep Q-Network (DQN) Agent with parameters  8: Initialize Meta-Model  9: Initialize experience replay buffer $\mathcal{B\leftarrow\emptyset}$  10: for episode = 1 to N do  11: Reset environment and get initial state $s_{0}= \left[ c_{TP}\left( z_{1}^{0} \right),c_{FN}\left( z_{2}^{0} \right) \right]$  12: for k = 1 to K do  13: Agent selects action $\text{ }a_{k}$based on state $s_{k}$:  14: $a_{k}=\left[ \Sigma_{PP}\left( \Delta_{PN} \right)_{SP}\left( k \right) \right]\leftarrow\text{DQN Select Action}\left( s_{k} \right)$  15: Apply action to adjust constraints:  16: $c_{TP}(z_{1}^{k+1})=c_{TP}(z_{1}^{k})+\Delta_{TP}(k)$  17: $c_{FN}(z_{2}^{k+1})=c_{FN}(z_{2}^{k})+\Delta_{FN}(k)$  18: Run ADMM Optimization with updated constraints:  19: $\left( P_{c}^{k+1},P_{d}^{k+1} \right)=ADMM\left( f,c_{TP},c_{FN},\rho,P_{c}^{k},P_{d}^{k},z_{1}^{k},z_{2}^{k},d^{k},y_{1}^{k},y_{2}^{k},y_{3}^{k},K,\epsilon\right)$  20: valuate detection performance:  21: Compute F1 score and other metrics based on$\left( P_{c}^{k+1},P_{d}^{k+1} \right)$  22: Compute reward:  23: $r_{k}=w_{1}\cdot F1-w_{2}\cdot(\Delta_{TP}(k)^{2}+\Delta_{FN}(k)^{2})+w_{3}\cdot\alpha▹\alpha\text{ measures the balance between falsepositives and false negatives}$  24: Store experience in replay buffer:  25: $\mathcal{B\leftarrow B\cup\{}\left( s_{k},a_{k},r_{k},s_{k+1} \right)\}$  26: Sample minibatch from $\mathcal{B}$ and train DQN:  27: DQN$\_$Train $\mathcal{(B)}$  28: Extract sample features and parameter strategies from $\left( P_{c}^{k+1},P_{d}^{k+1} \right)$  29: Meta$\_$Model$\_$Train (features$,\left[ \Delta_{TP}\left( k \right),\Delta_{FN}\left( k \right) \right])$  30: Update state:  31: $s_{k+1}=\left[ c_{TP}\left( z_{1}^{k+1} \right),c_{FN}\left( z_{2}^{k+1} \right) \right]$  32: Check convergence:  33: if primal residual< $\epsilon$ and dual$\_$residual< $\epsilon$ then  34: Terminate inner loop  35: end if  36: end for  37: Decay exploration rate of DQN  38: end for |
| 39: Return:$P_{c}^{*},P_{d}^{*}$  40: Combine the Meta Features and $\{P_{c}^{*},P_{d}^{*}\}$ to construct the meta-dataset  41: $MetaData=\{\left( fea_{1},P_{c}^{*},P_{d}^{*} \right),\left( fea_{2},P_{c}^{*},P_{d}^{*} \right),\ldots,\left( fea_{n},P_{c}^{*},P_{d}^{*} \right).$  42: Trained Meta-Model |

1. **Meta-model Training**

In panel-based targeted sequencing, genomic regions of interest are typically predefined and listed explicitly in a BED (Browser Extensible Data) file. A BED file is a tab-delimited text file specifying genomic intervals, each represented by chromosome name, start, and end positions. In practice, these intervals indicate specific genomic regions targeted during sequencing, such as coding regions of cancer-related genes or known mutation hotspots. Each line of the BED file corresponds to one such target region.

Given the inherent heterogeneity of ctDNA samples, MRDagent leverages the BED file as a guide to systematically divide the genome into meaningful sub-regions for subsequent analysis. Specifically, we segment the genomic data (aligned sequencing data stored in BAM files) according to the intervals provided in the BED file. These sub-regions represent distinct genomic intervals exhibiting potentially unique variant characteristics due to biological and technical variability.

MRDagent then extracts these sub-regions for further analysis. Throughout the splitting process, meticulous care is taken to prevent breakpoints from occurring in mismatched regions, ensuring the consistency and accuracy of each sub-region. Subsequently, these sub-regions are systematically renamed and renumbered to create new BAM file subsets (e.g., BAM_Sub_1, BAM_Sub_2, …, BAM_Sub_N) and establish mapping relationships with the original sample indices. Additionally, the gold standard results in the VCF files of these ctDNA data are segmented according to the sub-regions and correspond one-to-one with the respective sub-regions.

After obtaining the sub-regions containing variants, we extract meta-features that distinguish the calling performance of parameter configuration within these sub-regions. Inspired by Wang et al. [1,2], we achieve this by extracting meta-features from the ctDNA data in BAM/SAM format files. Specifically, within the data segmentation module, we have integrated a fast-scanning algorithm, which differs from precise variant detection tools. This algorithm efficiently extracts meta-features by leveraging tools such as samtools, bcftools, and information from CIGAR, enabling rapid and effective feature extraction.

$$y^{*}=arg\min_{y}\left[ L\left( y,g\left( X \right) \right)+\lambda\cdot C\left( y,X \right) \right]$$

Where, $y^{*}$ represents the optimal predictive structure determined for a given input dataset $X$ . $y$ denotes a candidate predictive structure selected from the set of possible solutions. $\cdot$ $X$ is the input data or sample. $g\left( X \right)$ is the feature representation extracted from the input data $X$ by CNN. $L\left( y,g\left( X \right) \right)$ denotes the loss function that quantifies the deviation between the predicted structure $y$ and the ground truth or targets based on the extracted features $g\left( X \right).$ $C\left( y,X \right)$ is a regularization term designed to control the complexity of the predictive structure $y$, ensuring its consistency with historical data characteristics. $\lambda$ is a hyperparameter balancing the contributions of the loss function $L$ and the regularization term $C.$

Reference

1. Wang S, Liu Y, Wang J, et al. Is an SV caller compatible with sequencing data? An online recommendation tool to automatically recommend the optimal caller based on data features. Front Genet. 2023; 13: 1096797.
2. Wang S, Zhu X, Wang X, et al. TMBstable: a variant caller controls performance variation across heterogeneous sequencing samples. Brief Bioinform. 2024; 25(3): bbae159.
3. **Metrics to evaluate the detection performance and stability**

$$\begin{aligned} Sensitivity= \frac{TP}{TP+FN}\#\left( 16 \right) \end{aligned}$$

$$\begin{aligned} Precision= \frac{TP}{TP+FP}\#\left( 17 \right) \end{aligned}$$

$$\begin{aligned} F1 Score=2* \frac{Precision*Sensitivity}{Precision+Sensitivity}\#\left( 18 \right) \end{aligned}$$

Coefficient of Variation (CV)

The Coefficient of Variation (CV) is the ratio of the standard deviation to the mean, used to measure the relative variability of the data:

$$\begin{aligned} CV=\frac{\sigma}{\mu}\#\left( 8 \right) \end{aligned}$$

Where $\sigma$ is the standard deviation $\mu$ is the mean

Root Mean Square Error (RMSE)

RMSE is used to evaluate the error of a model, representing the square root of the mean squared differences between predicted values and actual values:

$$\begin{aligned} RMSE=\sqrt{\frac{1}{n}\sum_{i=1}^{n} \left( y_{i}-\hat{y_{i}} \right)^{2}}\#\left( 9 \right) \end{aligned}$$

Where n is the number of samples $y_{i}$ is the actual value $\hat{y}_{i}$ is the predicted value

1. **ctDNA datasets**

In this study, simulated ctDNA data were generated based on high-confidence somatic variant calls (single nucleotide variants [SNVs] and insertions/deletions [Indels]) extracted from the International Cancer Genome Consortium (ICGC) database (<https://platform.icgc-argo.org/>). After stringent quality filtering, a reliable variant dataset consisting of 22 SNVs and 46 Indels was obtained. For each simulated sample, we designed panels comprising between 40 genomic regions, each region with a length of 1 kb. The filtered SNV and Indel variants were distributed across these predefined panel regions. Using the GRCh38 human reference genome and a sequencing error model, we generated 400 simulated whole-exome sequencing (WXS) datasets via the GSDcreator tool (simulation details described in Supplementary Methods Section 4). Each simulated WXS dataset was then mixed with normal reference samples derived from GRCh38, diluting variant allele frequencies (VAFs) to realistic ctDNA detection levels ranging from 0.01% to 0.1%.

For the real-data analysis, we selected 27 prostate cancer WXS samples from the PACA-CA project (accessed via a DACO-authorised account at <https://platform.icgc-argo.org/>). Each original sample was segmented into multiple 1 kb genomic panels. Subsequently, we performed downsampling using Samtools to simulate lower coverage sequencing. The resulting tumor panels were then merged with normal reference samples (GRCh38), ensuring final VAFs within the 0.01%–0.1% range. This procedure yielded a total of 1,753 ctDNA genomic regions **(listed in Supplementary Table 1)**, which were further grouped into 473 sub-samples based on comparable VAF distributions **(detailed in Supplementary Table 2)**.

1. **Execution details for each variant detection tool**

**Mutect2:**

**(1) Background:**

Mutect2 is developed by the Broad Institute and released as part of GATK4 in 2018. It is specifically designed to call somatic single-nucleotide variants (SNVs) and small indels from paired tumor–normal sequencing data. It incorporates community best practices—including use of a panel of normals (PON), gnomAD germline resource, and contamination estimation—to reduce false positives. Mutect2 has become a standard in large cancer genome projects such as TCGA and ICGC.

**(2) Basic Principles:**

1. Local de Bruijn Graph Assembly: For each candidate region, reads are assembled into haplotypes via a de Bruijn graph, enabling detection of complex indels and nearby SNVs.
2. Pair-HMM Likelihoods: Reads are aligned to each assembled haplotype using a Pair Hidden Markov Model to compute per-read likelihoods.
3. Bayesian Somatic Genotyping: Combines tumor and normal likelihoods with priors on allele fraction, computing a log-odds score (LOD) for somatic vs. germline or noise.
4. Filtering Module: Applies filters such as strand bias, orientation bias (using a trained model), read position bias, and contamination estimate to flag false positives.

*Mutect2*

*Mutect2 from GATK v4.2.0.0 was obtained from https://github.com/broadinstitute/gatk. Variants were called using: gatk Mutect2 --reference <ref.fa> --input <sample.bam> --output <output_dir>/mutect2.vcf.gz --tumor-lod-to-emit 3.0 --af-of-alleles-not-in-resource 0.0000025 --germline-resource <germline_resource.vcf.gz> --panel-of-normals <pon.vcf.gz>.*

*Only variants marked as PASS were accepted.*

References:

Benjamin D. et al. (2019) Calling somatic SNVs and indels with Mutect2. BioRxiv, 861054.

**Freebayes:**

**(1) Background:**

FreeBayes was first published by Erik Garrison et al. in 2012 as a haplotype-based Bayesian variant caller. It handles multi-sample, multi-allelic contexts and is widely used for population genetics, cancer, and microbial studies. Unlike site-by-site callers, FreeBayes operates on “active regions” to jointly consider overlapping reads and haplotype structure.

**(2) Basic Principles:**

1. Active Region Detection: Scans the genome to identify regions with evidence of variation (e.g., mismatches, indels).
2. Read Clustering into Haplotypes: Groups reads that share similar variant patterns and enumerates possible haplotypes within each region.
3. Bayesian Genotype Inference: For each sample and locus, computes posterior probabilities of genotypes given observed reads and allele-frequency priors.
4. Multi-Allelic and Multi-Sample Model: Simultaneously handles more than two alleles per locus and multiple samples, improving sensitivity in complex scenarios.

*Freebayes*

*Freebayes v1.3.2 was obtained from https://github.com/freebayes/freebayes. Variants were called using:freebayes -f <ref.fa> -v <output_dir>/freebayes.vcf.gz --ploidy 2 <sample.bam>.*

*Only variants with QUAL > 20 were accepted.*

References:

Garrison E. et al. (2012) Haplotype-based variant detection from short-read sequencing. *arXiv preprint*, arXiv:1207.3907.

**LoFreq:**

**(1) Background:**

LoFreq was introduced by Wilm et al. in 2012 to detect low-frequency variants (<1%) in viral and microbial deep sequencing data. It leverages base-call quality scores for error correction and achieves high specificity in high-noise contexts. LoFreq is now also applied in circulating tumor DNA (ctDNA) and ultra-deep cancer panels.

**(2) Basic Principles:**

1. Base Quality Recalibration: Adjusts each base’s Phred quality score to correct systematic biases before variant calling.
2. Per-Base Error Modeling: Estimates position-specific error rates from recalibrated quality scores.
3. Poisson–Binomial Statistical Test: For each site, models number of non-reference reads under the background error distribution and computes a p-value for true variant presence.
4. Multiple-Testing Correction: Applies Benjamini–Hochberg or similar to control false discovery rate across all tested sites.

*LoFreq*

*LoFreq v2.1.5 was obtained from https://csb5.github.io/lofreq/. Variants were called using:*

*lofreq indelqual --dindel -f <ref.fa> -o <output_dir>/sample.indelqual.bam <sample.bam>*

*lofreq call-parallel --pp-threads 16 -f <ref.fa> -o <output_dir>/lofreq.vcf.gz <output_dir>/sample.indelqual.bam.Only variants with QUAL > 20 were retained.*

References:

Wilm A. et al. (2012) LoFreq: a sequence-quality aware, ultra-sensitive variant caller for uncovering cell-population heterogeneity from high-throughput sequencing datasets. *Nucleic Acids Research*, 40(22), 11189–11201.

**VarScan2:**

**(1) Background:**

VarScan2, published by Koboldt et al. in 2012, is one of the earliest tools for somatic mutation calling. It supports both paired tumor–normal analyses and single-sample calling. VarScan2 is valued for its transparency, offering straightforward pileup-based statistics and robust filtering options. It has been applied extensively in TCGA, TARGET, and many clinical sequencing studies.

**(2) Basic Principles:**

1. Pileup Generation: Uses SAMtools mpileup to count reference and non-reference bases, read depth, and strand orientation at each position.
2. Statistical Testing: Compares tumor vs. normal base counts via Fisher’s exact test (or χ²) to identify somatic SNVs/indels.
3. Allele Frequency and Depth Thresholds: Requires user-defined minimum variant allele frequency and minimum depth in both tumor and normal to filter calls.
4. Annotation and Filtering: Outputs VCF with fields like “SSC” (somatic score) and supports downstream scripts for further filtering (e.g., strand bias, read position).

*VarScan2*

*VarScan2 v2.4.4 was obtained from https://sourceforge.net/projects/varscan/. Variants were called using:samtools mpileup -f <ref.fa> <sample.bam> > <output_dir>/mpileup.txt*

*java -jar VarScan.jar mpileup2snp <output_dir>/mpileup.txt --min-var-freq 0.01 --output-vcf 1 > <output_dir>/varscan2.vcf.*

*Variants with a minimum coverage of 10 and QUAL > 20 were accepted.*

References:

Koboldt D.C. et al. (2012) VarScan 2: somatic mutation and copy number alteration discovery in cancer by exome sequencing. *Genome Research*, 22(3), 568–576.

**Bcftools (mpileup + call):**

**(1) Background:**

Bcftools, maintained by Heng Li’s team since around 2010, is a versatile toolkit for VCF/BCF manipulation, filtering, and variant calling. Its “mpileup + call” pipeline provides a lightweight variant caller suitable for germline and simple somatic analyses. Bcftools emphasizes speed and memory efficiency, making it ideal for large cohorts.

**(2) Basic Principles:**

1. mpileup Generation: Summarizes per-base read counts, base qualities, mapping qualities across samples.
2. Likelihood Model: Calculates for each candidate allele the log-likelihood ratio (LLR) comparing alternative vs. reference, using an approximate Bayesian or maximum-likelihood framework.
3. Allele Frequency Priors: Integrates priors on allele frequency (e.g., Hardy–Weinberg for germline) to refine calls.
4. Variant Calling and Filtering: Produces VCF with genotype likelihoods (GL) or Phred-scaled quality (QUAL) scores, and supports rich filtering expressions on INFO/FORMAT fields.

*Bcftools*

*Bcftools v1.14 was obtained from https://samtools.github.io/bcftools/. Variants were called using:bcftools mpileup -f <ref.fa> <sample.bam> | bcftools call -mv -Oz -o <output_dir>/bcftools.vcf.gz.Only variants with QUAL > 20 were retained.*

References:

Danecek P. et al. (2021) Twelve years of SAMtools and BCFtools. *GigaScience*, 10(2), giab008.

**SiNVICT:**

**(1) Background:**

SiNVICT was introduced to enable robust detection of single-nucleotide variants (SNVs) and small indels in circulating tumour DNA (ctDNA) sequencing data, even at very low variant allele frequencies (<0.5%). It was developed to overcome challenges inherent to liquid biopsy — notably the mixture of normal and tumour-derived DNA, heterogeneous subclone representation, and high background noise from sequencing and mapping errors. SiNVICT integrates platform-specific error modelling, position- and context-aware filters, and the ability to jointly analyse multiple samples (including time-series data) to achieve both high sensitivity and specificity in ultra-deep sequencing contexts

**(2) Basic Principles:**

1. Pre-Processing & Error Correction: Reads are trimmed to remove primers and low-quality bases, then aligned using an indel-aware mapper (e.g., BWA or mrFAST-fastHASH). Base quality scores are recalibrated, and local assembly (via ABRA) corrects ambiguous mappings and systematic errors. Per-base metrics are extracted with bam-readcount .
2. Poisson-Based Variant Nomination:
3. Mutation Presence Test (p₁): The number of variant-supporting reads (K) at depth N is compared against the platform’s average error rate r using a Poisson cumulative distribution, yielding a P-value for true mutation versus noise.
4. Somatic vs. Germline Test (p₂): A second Poisson test assumes ~N/2 variant reads for heterozygous germline variants to distinguish somatic events. Quality scores (Q = –10 log₁₀ P) threshold initial calls .
5. Post-Processing & Noise Filtering: Loci below a minimum read-depth threshold are discarded. Strand bias is assessed by the forward-strand read ratio, with calls outside a narrow window around 0.5 removed. Homopolymers (≥3 identical bases) adjacent to the site trigger additional filtering. In cohort or time-series analyses, variant allele frequencies across samples are used to compute a signal-to-noise ratio (mean VAF/SD), filtering out recurrent noise hotspots .
6. Optional Time-Series Analysis: High-confidence loci present in all time points are re-evaluated using localized error rates derived from neighboring bases, allowing sensitive tracking of clonal dynamics over treatment courses

*SiNVICT*

*SiNVICT v1.2.0 was obtained from https://github.com/AstraZeneca-NGS/SiNVICT. Variants were called using:sinvict --bam <sample.bam> --ref <ref.fa> --output <output_dir>/sinvict.vcf.gz --threads 16.*

*Only variants marked as PASS were accepted*

References:

Kockan C. et al. (2017) SiNVICT: ultra-sensitive detection of single nucleotide variants and indels in circulating tumour DNA. *Bioinformatics*, 33(1), 26–34.

**PACT:**

1. **Background:**

PACT (“Pipeline for the Analysis of ctDNA”) is an open‐source, unified workflow designed to detect small mutations (SNVs/indels), copy-number alterations (CNAs), and structural variants (SVs) from circulating tumor DNA (ctDNA) sequencing data. Developed at Washington University in St. Louis, it addresses key challenges in ctDNA analysis—low tumor fraction (<1 % VAF), deep sequencing noise, and the need to distinguish somatic events from germline or technical artifacts—by integrating multiple callers, matched normal controls, and a panel of unmatched healthy controls in a Common Workflow Language (CWL) pipeline.

1. **Basic Principles:**
2. Ensemble Candidate Nomination: PACT first runs a suite of specialized callers for SNVs, CNAs, and SVs using relaxed filtering criteria to maximize sensitivity, producing an initial list of candidate variants .
3. Normalization & Noise Filtering: All nominated variants undergo normalization and statistical filtering tailored to the noise characteristics of ultra-deep ctDNA sequencing (e.g., low VAF, PCR/sequencer errors) .
4. Control-Based Filtering: Each candidate is genotyped against both a matched germline control and an unmatched panel of normals. Variants present in controls are flagged as germline or technical artifacts and removed .
5. Region-Specific SV Filtering: For structural variants, PACT enforces that at least one breakpoint lies within the user’s targeted capture regions, and excludes calls from known high–false-positive genomic loci .
6. Evidence Integration for SVs: Final SV calls must be supported by both split-read and discordant paired-end read evidence, ensuring robust breakpoint resolution .
7. Unified Reporting & Workflow Management: Outputs include per-variant VCFs and comprehensive QC reports (HTML/PDF) with sensitivity estimates, coverage metrics, and filtering summaries. The entire pipeline is packaged as a CWL workflow for ease of deployment on HPC or cloud systems.

*PACT*

*PACT v1.0.0 was obtained from https://github.com/Illumina/PACT. Variants were called using:pact --input <sample.bam> --reference <ref.fa> --output <output_dir>/pact.vcf.gz --threads 16.Variants with a minimum allele frequency of 0.01 and marked as PASS were retained.*

References:

Webster J. et al. (2023) PACT: a pipeline for analysis of circulating tumor DNA. *Bioinformatics,* 39(8), btad489.

**Platypus:**

1. **Background:**

Platypus, presented by Rimmer et al. in 2014, is a hybrid variant caller that jointly performs local assembly and Bayesian genotyping. It excels at small indel detection in both germline and somatic settings, and supports multi-sample calling. Platypus is embedded in pipelines for whole-genome, exome, and targeted sequencing in human and model organisms.

**(2) Basic Principles:**

1. Joint Local Assembly: Simultaneously assembles reads from all samples in a locus into candidate haplotypes using a de Bruijn graph.
2. Per-Haplotype Likelihoods: Aligns reads to each haplotype with a Pair-HMM to compute likelihoods per sample.
3. Bayesian Multi-Sample Genotyping: Uses a Bayesian model that integrates haplotype likelihoods and population priors to call variants across samples.
4. High-Confidence Filtering: Applies filters for read depth, strand bias, mapping quality, and local sequence complexity to ensure accuracy.

*Platypus*

*Platypus v0.8.1 was obtained from https://github.com/andyrimmer/Platypus. Variants were called using:Platypus.py callVariants --bamFiles=<sample.bam> --refFile=<ref.fa> --output=<output_dir>/platypus.vcf --minPosterior=20.Only variants with QUAL > 20 were retained.*

References:

Rimmer A. et al. (2014) Integrating mapping-, assembly- and haplotype-based approaches for calling variants in clinical sequencing applications. *Nature Genetics*, 46(8), 912–918.

**Supplementary Tables**

Supplementary Table S1

preliminary variant detection step parameter configurations

| Parameter | Explanation | Range | Type |
| --- | --- | --- | --- |
| base-quality-score-threshold (T) | Minimum base quality score required for a base to be considered. | (6, 25) | Discrete |
| callable-depth (T) | Minimum read depth required to call a site. | (5, 101) | Discrete |
| f1r2-median-mq (T) | Median mapping quality of F1R2 reads. | (30, 71) | Discrete |
| f1r2-min-bq (T) | Minimum base quality of F1R2 reads. | (6, 31) | Discrete |
| max-reads-per-alignment-start (T) | Maximum number of reads allowed per alignment start. | (0, 5001) | Discrete |
| pcr-indel-qual (T) | Quality score threshold for PCR indel filtering. | [10, 60] | Discrete |
| pcr-snv-qual (T) | Quality score threshold for PCR SNV filtering. | [10, 60] | Discrete |
| assembly-region-padding (T) | Padding size added to assembly regions. | (50, 2001) | Discrete |
| kmer-size (first instance) (T) | Size of kmers used in assembly (small regions). | (5, 25) | Discrete |
| kmer-size (second instance) (T) | Size of kmers used in assembly (large regions). | (25, 50) | Discrete |
| max-assembly-region-size (T) | Maximum size of assembly regions. | (200, 001) | Discrete |
| max-prob-propagation-distance (T) | Maximum distance for probability propagation. | (40, 301) | Discrete |
| min-assembly-region-size (T) | Minimum size of assembly regions. | (30, 151) | Discrete |
| max-unpruned-variants (T) | Maximum number of unpruned variants allowed. | (50, 501) | Discrete |
| min-dangling-branch-length (T) | Minimum length of dangling branches allowed in assembly graph. | (2, 10) | Discrete |
| phred-scaled-global-read-mismapping-rate (T) | Phred-scaled global mismapping rate threshold for reads. | (30, 51) | Discrete |
| pair-hmm-gap-continuation-penalty (T) | Penalty for gaps in PairHMM calculations. | (6, 15) | Discrete |
| mbq (T) | Minimum base quality for read bases to be considered. | (6, 20) | Discrete |
| init-lod (X) | Initial LOD threshold for variant calling. | (0.5, 3.5) | Continuous |
| max-af (X) | Maximum allowed allele frequency for variants. | (0.005, 0.05) | Continuous |
| emit-lod (X) | LOD threshold for emitting potential variants. | (1.5, 3.0) | Continuous |
| active-probability-threshold (X) | Probability threshold for activating a region. | (0.0005, 0.01) | Continuous |
| adaptive-pruning-initial-error-rate (X) | Initial error rate for adaptive pruning in assembly. | (0.0005, 0.005) | Continuous |
| pruning-lod-threshold (X) | LOD threshold for pruning assembly graph. | (2, 5) | Continuous |
| flow-probability-threshold (X) | Threshold for flow-based probability. | (0.002, 0.01) | Continuous |
| expected-mismatch-rate-for-read-disqualification (X) | Expected mismatch rate for disqualifying reads. | (0.01, 0.05) | Continuous |
| min-AF (X) | Minimum allele frequency threshold for variant calling. | (1e-5, 1e-3) | Continuous |

Explanation:

Discrete parameters (T): These parameters only take integer values within the specified ranges, or specific lists of integer values.

All T parameters correspond to bound_T, which means they are constrained to integer sets, either in ranges or predefined lists.

Continuous parameters (X): These parameters can take any real value within the specified ranges. They correspond to bound.

These parameters typically control thresholds and probabilities in variant calling and filtering workflows.

Supplementary Table S2

Filtering step parameter configurations

| Parameter | Explanation | Range | Type |
| --- | --- | --- | --- |
| distance_on_haplotype (X) | Maximum allowable distance between variants on the same haplotype for consideration. | (10, 200) | Continuous |
| f_score_beta (X) | Weighting factor for precision-recall balance in F1-score calculation. | (0.5, 2.0) | Continuous |
| false_discovery_rate (X) | Threshold for controlling the expected proportion of false discoveries among the identified variants. | (0.001, 0.2) | Continuous |
| initial_threshold (X) | Initial confidence threshold for variant calling. | (0.005, 0.2) | Continuous |
| log_artifact_prior (X) | Log-scaled prior probability of artifacts in sequencing data. | (-5.0, -1.5) | Continuous |
| log_indel_prior (X) | Log-scaled prior probability of indels in sequencing data. | (-20.0, -10.0) | Continuous |
| log_snv_prior (X) | Log-scaled prior probability of single-nucleotide variants (SNVs) in sequencing data. | (-20.0, -10.0) | Continuous |
| max_events_in_region (T) | Maximum number of variant events allowed in a specific genomic region. | (2, 10) | Discrete |
| min_slippage_length (T) | Minimum length of homopolymer repeats required to identify PCR slippage events. | (4, 12) | Discrete |
| pcr_slippage_rate (X) | Estimated rate of PCR slippage in sequencing data. | (0.005, 0.3) | Continuous |

Explanation:

Discrete parameters (T): These parameters only take integer values within the specified ranges, or specific lists of integer values.

All T parameters correspond to bound_T, which means they are constrained to integer sets, either in ranges or predefined lists.

Continuous parameters (X): These parameters can take any real value within the specified ranges. They correspond to bound.

These parameters typically control thresholds and probabilities in variant calling and filtering workflows.

Supplementary Table S3

Meta-feature Explanation

| Meta Feature | Explanation |
| --- | --- |
| Reference Allele Count | The count of reads that support the reference allele at the variant site. |
| Non-reference Allele Count | The count of reads that support the non-reference (alternative) allele at the variant site. |
| Sum of Base Qualities | The sum of base quality scores for all reads at the variant site, indicating sequencing confidence. |
| Tumour Coverage | The total number of reads covering the variant site in tumour samples. |
| Mapping Quality | The average mapping quality of reads at the variant site, reflecting alignment accuracy. |
| Median Read Position | The median position of the variant within the sequencing reads, used to assess positional bias. |
| Homopolymer Rate | The proportion of homopolymer regions (repeated bases) near the variant site. |
| GC Content | The percentage of guanine (G) and cytosine (C) nucleotides in the surrounding sequence. |
| Trinucleotide Sequence Counts | The frequency of specific trinucleotide sequences near the variant site. |
| Trinucleotide Total Count | The total count of trinucleotide sequences in the region surrounding the variant site. |
| Distance to Germline SNP | The distance between the detected variant and the nearest known germline single nucleotide polymorphism (SNP). |
| Repeat Percentage | The proportion of repeated sequences in the region around the variant site. |
| Short SV Percentage | The percentage of short structural variants (SVs) in the region around the variant. |
| Middle SV Percentage | The percentage of medium-sized structural variants (SVs) in the region around the variant. |
| Long SV Percentage | The percentage of long structural variants (SVs) in the region around the variant. |
| Average Read Length | The average length of sequencing reads covering the variant site. |
| Small Gap Account | The number of small gaps (insertions or deletions) near the variant site. |
| RMB (Read Mismatch Bias) | The bias in the occurrence of mismatched reads at the variant site. |
| HMDP (High-Quality Mismatch Density Percentage) | The density of high-quality mismatches in the surrounding sequence. |
| Average Depth | The average sequencing depth at the variant site. |
